# Leaf rolling and leaf angle improve fog capturing and transport in wheat; adaptation for drought stress in an arid climate

**DOI:** 10.1186/s40529-022-00343-y

**Published:** 2022-05-16

**Authors:** Sabah Merrium, Zulfiqar Ali, Muhammad Habib-ur-Rahman, Sadia Hakeem, Muhammad Arslan Khalid

**Affiliations:** 1 Institute of Plant Breeding and Biotechnology, MNS-University of Agriculture, Multan, 60000 Pakistan; 2 Institute of Crop Science and Resource Conservation (INRES), Crop Science Group, University of Bonn, Bonn, Germany; 3 Department of Plant Breeding and Genetics, University of Agriculture Faisalabad, Faisalabad, Pakistan

**Keywords:** Leaf repellency, Phenotyping, Morpho-physiological traits, Physiological parameters, Contact angle, Leaf wettability, Changing climate

## Abstract

**Background:**

Plants use different mechanisms to transport the collected fog water. Leaf traits of wheat play an important role in directing fog water through leaf rolling and leaf angle into the root zone, where it can be stored for consumption. Wheat leaf traits can enhance fog capturing under drought stress. To examine this, 200 wheat genotypes were characterized for leaf rolling and leaf angle under optimal conditions in the field using a randomized complete block design. Seven different phenotypic combinations for leaf traits were observed. A core set of 44 genotypes was evaluated under drought stress.

**Results:**

Results show that variability for leaf traits existed among genotypes. An association was found between leaf rolling and leaf angle, moisture capturing, physiological parameters, and yield contributing traits using correlation. Physiological parameters, especially water use efficiency, were positively correlated with grain yield and moisture capturing at both growth stages. The genotypes (G11 at tillering and G24 at booting phonological phases) with inward to twisting type rolling and erect to semi-erect leaf angle capture more water (12–20%) within the root zone. Twenty-one genotypes were selected based on moisture capturing efficiency and evaluated for leaf surface wettability. Association was found between fog capturing and wettability. This shows that it was due to the leaf repellency validated from static contact angle measurements.

**Conclusion:**

These results will give insights into fog capturing and the development of drought-tolerant crops in the semi-arid and arid regions.

**Supplementary Information:**

The online version contains supplementary material available at 10.1186/s40529-022-00343-y.

## Introduction

Leaf wettability is the most common phenomenon in the plants through which water retention on the leaf surface occurred as a result of fog, dew, and gaseous/atmospheric water. Leaf wetting has both positive and negative effects on plant growth and development. Wettability of the leaf surfaces can have a positive effect by supplying water through water absorption or transpiration suppression and improve plant water status (Eller et al. 2013; Goldsmith et al. 2013; Gotsch et al. 2014; Ali et al. 2022). Alternatively, negative effects inhibit leaf gas exchange, nutrients leaches, and promote pathogen growth on the leaf surfaces (Bradley et al. 2003; Sase et al. 2008). Negative effects lead to the identification of the traits that led to the water repellency on the leaf surface (Rosado and Holder 2013). Traits associated with leaf surface, morphology, and architecture are positively correlated with leaf wettability and also vary based on the climate variables (Berry et al. 2019).

Fog, dew, and atmospheric water are the sustainable source of water that is utilized by the plants. Plants use these sources efficiently by using their leaf surface structure and morphology that increased the leaf wetness (Azad et al. 2015; Gürsoy et al. 2017). Leaf surface wettability consists of two mechanisms i.e. water absorption and water repellency. Water is absorbed in the plant species through stomata, veins, trichomes, and cuticles *i.e., Sequoia sempervirens* (Bahamonde et al. 2018; Burkhardt et al. 2012; Fernández et al. 2016). Similarly, water repellency is due to thick cuticles, waxes, and glabrous surfaces *i.e., D. Draco* (Klimko and Wiland-Szymanska 2008).

Due to the frequency and intensity of drought stress in many regions of the world, it is predicted that water availability for the growing of wheat crops will decrease (Zhao et al. 2017). Under this scenario, the water supply for the wheat plant is needed to increase by using atmospheric water resources and leaf wettability mechanisms for sustainable production. Wheat is a major cereal crop of Pakistan and also important as a food crop (Ul Hassan et al. 2021; Rahman et al. 2021a). It is grown in the areas that are affected by water shortage (Hussain et al. 2021). Multan also includes in these regions where wheat crops face dry periods and fog is a common occurrence, especially during December, January, and February (Rahman et al. 2018, 2021b; Ghafoor et al. 2021). Fog persists in this region due to low temperature at night and high humidity (Additional file 2: Fig. S1). Leaf wetting has positive effects on plant water status, and wheat plants have leaf morphology that may help in the nucleation of fog water (Azad et al. 2015; Berry et al. 2019; Roth-Nebelsick et al. 2012; Sharma et al. 2018, 2016). For example, in wheat, morphological traits, leaf rolling and leaf to stem angle can suppress the transpiration and also help to move the water toward the root zone due to leaf surface repellency (Rosado and Holder 2013). In wheat, leaf rolling dynamics have the potential to capture fog water and leaf angle allows the captured water to pin down the water droplets in the root zone similar to *Stipagrostis sabulicola* (Ju et al. 2012; Roth-Nebelsick et al. 2012). These morphological structures can be architectured genetically to promote the fog capturing of a wheat plant.

Here we studied how leaf traits and morpho-physiological traits are associated with fog capturing, specifically the moisture nucleation towards the root zone. Based on the leaf traits combination and moisture contents, we hypothesized that leaf rolling dynamics and leaf angle can capture fog water and transport it towards the root zone but leaf rolling is affected due to water stress. For this purpose, a core set of genotypes have been evaluated under drought conditions. The association of leaf surface traits with morpho-physiological traits and leaf wettability was determined. The results of these studies shed light on the role of fog capturing in wheat that can help the wheat breeder to improve plant features and drought tolerance.

## Methods and materials

### Plant material

The plant material comprised of 200 wheat genotypes collected from MNS University of Agriculture, Multan (Additional file 1: Table S1). Wheat genotypes were sown in the field at the Institute of Plant Breeding and Biotechnology, MNS University of Agriculture, Multan located at 30.08° latitude, 71.26° longitude, and 122 m elevation from sea level. It is situated in dry arid agro-climatic conditions. The historic climatic pattern in the Multan region for air temperature and precipitation were calculated by using long-term data (2008 to 2017) collected from Pakistan Metrological Department (Additional file 2: Fig. S1). Genotypes were sown in micro-plots consisting of a 1-m long row of each genotype under randomized complete block design in two replicates during the growing season 2017–2018. Each micro-plot had four genotypes. Genotypes were characterized visually after 60 DAS for leaf surface traits i.e. leaf erectness, longitudinal leaf rolling. Based on different phenotypic combinations, 72 genotypes were selected for the evaluation of moisture contents. Selected 44 genotypes, based on moisture contents were grown in plots of 1 m^2^ under randomized block design with split plot arrangement in three replicates in 2018–19. There were two treatments i.e. normal (4 irrigation including pre-sowing) and drought (1 irrigation at pre-sowing and 1 irrigation at tillering stage). Distance between plants and rows were maintained at 10 cm and 30 cm respectively. Recommended agronomic practices were applied.

### Measurement of leaf traits and morphological parameters

The germplasm was characterized visually for leaf rolling and leaf angles (Pask et al. 2012) at two distinct plant growth stages i.e., tillering and booting. Leaf-rolling was scored as 3 to 1 based on visual observation i.e., *Score* = 3 corresponded to inward leaf-rolling (when margins of the leaf rolled inward longitudinally from the ad-axial surface); *Score* = 2 corresponded to twisted type leaf-rolling, i.e. when the leaf margins were rolled inward ad-axially and outward ab-axially; *Score* = 1 corresponded to outward leaf rolling (when margins of the leaf in-rolled longitudinally from the ab-axial surface). Leaf angle/erectness was also scored using a visual scale from 4 to 1: *Score* = 4 corresponded to erect angle (when leaf to stem angle 30–40°); *Score* = 3 corresponded to semi-erect angle (when leaf to stem angle 40–60°); *Score* = 2 corresponded to semi droopy angle (when leaf to stem angle 60–90°), and *Score* = 1 corresponded to droopy angle (when leaf to stem angle 60–90°). Inclination between the leaf blade midrib and the stem was done by dividing the vertical plane into four sectors of approximately 30°. Five plants for each genotype were considered. Yield contributing traits including flag leaf traits (flag leaf length, width, and area), plant height, days to heading and maturity, peduncle length, ear length, spike weight, spikelets per spike, seeds per ear, and grain yield per plot were recorded. Three measurements from each genotype and replication were made and average were taken.

### Climatic conditions

Monthly records of air and soil temperature (^o^C), rainfall (mm), relative humidity (%), leaf wetness (wet and dry minutes), wind speed (m s^−1^), wind direction (degree), and solar radiation (MJ m^−2^) were obtained from an automatic weather station (AWS) with data logger CR1000X (Campbell Scientific Inc. USA) that was installed near the study sites (1–3 m). The recordings were read using PC200W software. Variables were logged at 1 h intervals throughout the growing season. Climograph (Additional file 2: Fig. S2) shows that fog occurred during 12 AM and 8 AM, during the night and early morning in the Multan. It shows that relative humidity was maximum (> 75%) during night and minimum (> 45%) during the day except for April. Leaf wetness also showed that there was a continuous increase (> 50 min) in the number of wet hours during the night (12 AM to 10 AM) while leaf dryness started to increase after 10 AM to 6 PM and then decreased. It shows that fog events occurred during December, January, and February. Relative humidity and leaf wetness were maximum (more than 80% and 45 min respectively) during these months. The temperature also fluctuated between 10–15 °C minimum during night-time and 22–25 °C maximum during the daytime. Soil temperature shows the same trend as air temperature. Soil temperature fluctuated between 10–25 °C during night and day.

### Measurement of moisture contents

**Fig. 1:**
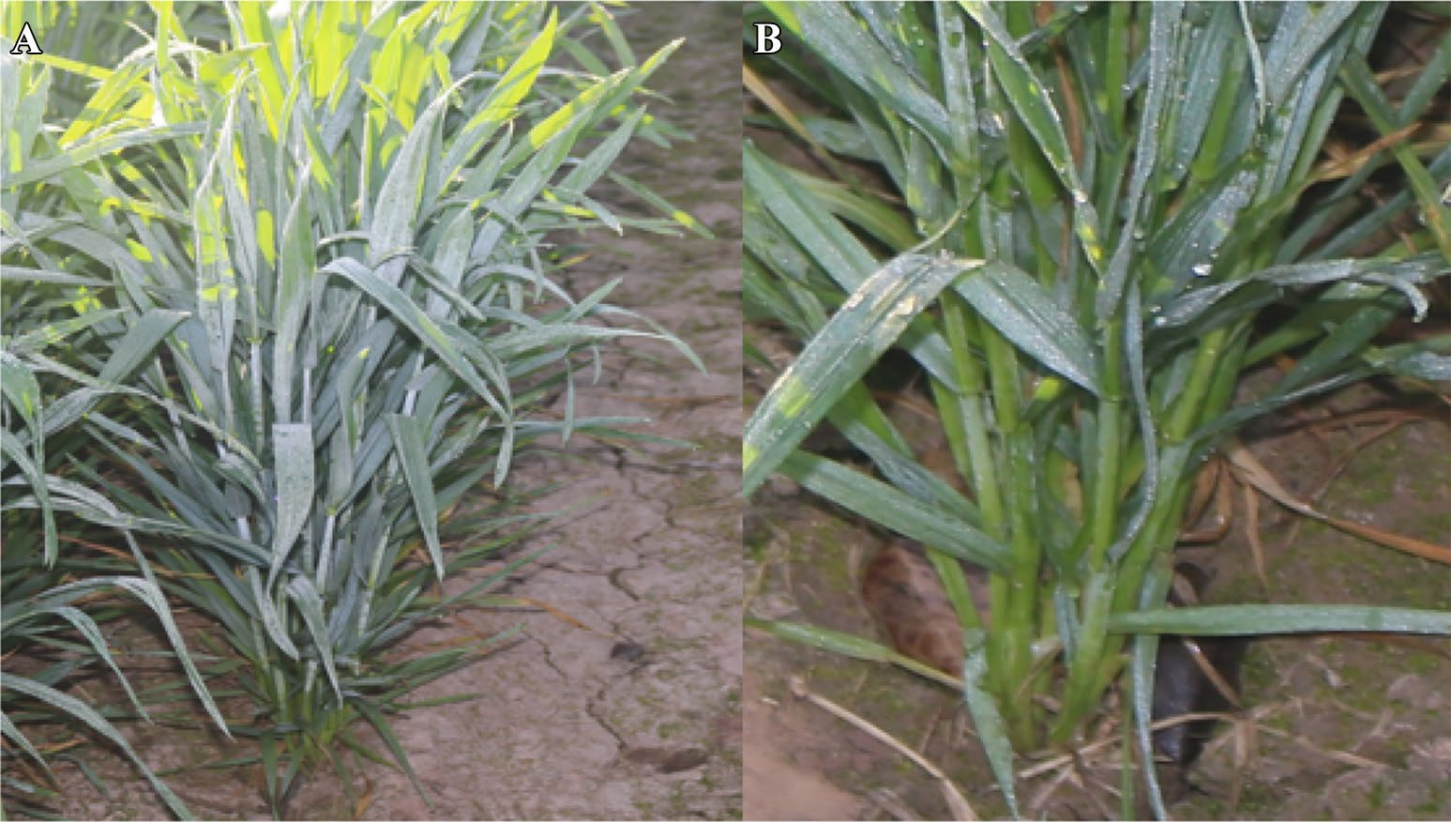
Photographs of **a** wheat plant during fog events and **b** dampness due to fog water input

During fog events, it was observed that the upper soil of each plant was damp due to the fog water input (Fig. 1). For the measurement of fog water input, moisture contents within root-zone (2 cm away from roots of the plant) and vicinity (30 cm apart from plant canopy) were recorded by using a portable soil moisture meter (WET-2; Delta-T devices). The moisture sensor was connected to HH2 read-out device and determined soil moisture content based on the dielectric properties of the soil (Delta-T devices 2007). A probe length of 68 mm was used for the measurement of moisture contents. The probe was also calibrated by using the gravimetric method. Three measurements from each genotype were recorded and averages were taken.

Moisture capturing was calculated as.

$$Moisture\,capturing\,\left(MC\right)=moisture\,percentage\,of\,the\,root\,zone\,(RZ)-moisture\,percentage\,of\,the\,vicinity (V)$$

### Measurement of physiological parameters

Physiological parameters of selected genotypes were recorded at the booting stage. The activity of physiological parameters namely stomatal conductance (mmol H_2_O m^−2^ s^−1^), photosynthesis (µmol CO_2_ m^−2^ s^−1^), transpiration (mmol H_2_O m^−2^ s^−1^), and water use efficiency (mmol CO_2_ mol^−1^ H_2_O) of the leaf plants, were recorded by using a CIRAS 3-Portable Photosynthesis System (CIRAS-3, PP Systems, Amesbury, USA) during mid-day (11:00 am to 2:00 pm). Mid portion of the top three leaves were used for data recording. Three observations were made from each genotypes and an average were taken.

### Leaf surface wettability

To measure the wettability of leaf surface 21 genotypes were selected based on phenotypic combinations. The static contact angle of the genotypes was measured at the stem elongation stage by using the contact angle device (Model OCA 25, data-physics instruments GmbH, Germany). The volume of the 3ul at a dosing rate of 0.5 µl/s was disposed on the adaxial and abaxial leaf surface for the measurement of static contact angle at room temperature. Five readings were recorded at different points and the average was calculated.

### Statistical analysis

Genotypic frequencies for characterization of leaf surface traits were determined by using R.3.6.1 Software. Bar plots with standard error bars were constructed by using ggplot2 package in R. For estimates of genetic parameters of leaf traits variability, the variability package of R was used by Raj Popat et al. (2020). Mean data of different traits were used to make biplots. Center and scale transformation were applied to data and biplots were created by using the ggbiplot package of R software (Vu 2016). Hierarchical clustering was also done by using the Euclidean method in R.3.6.1. The association of leaf surface traits with moisture capturing, physiological parameters, and yield contributing traits was assessed by using package cor and corplot in R software (Wei et al. 2017).

## Results

### Phenotypic variation of leaf rolling and leaf angle

**Fig. 2:**
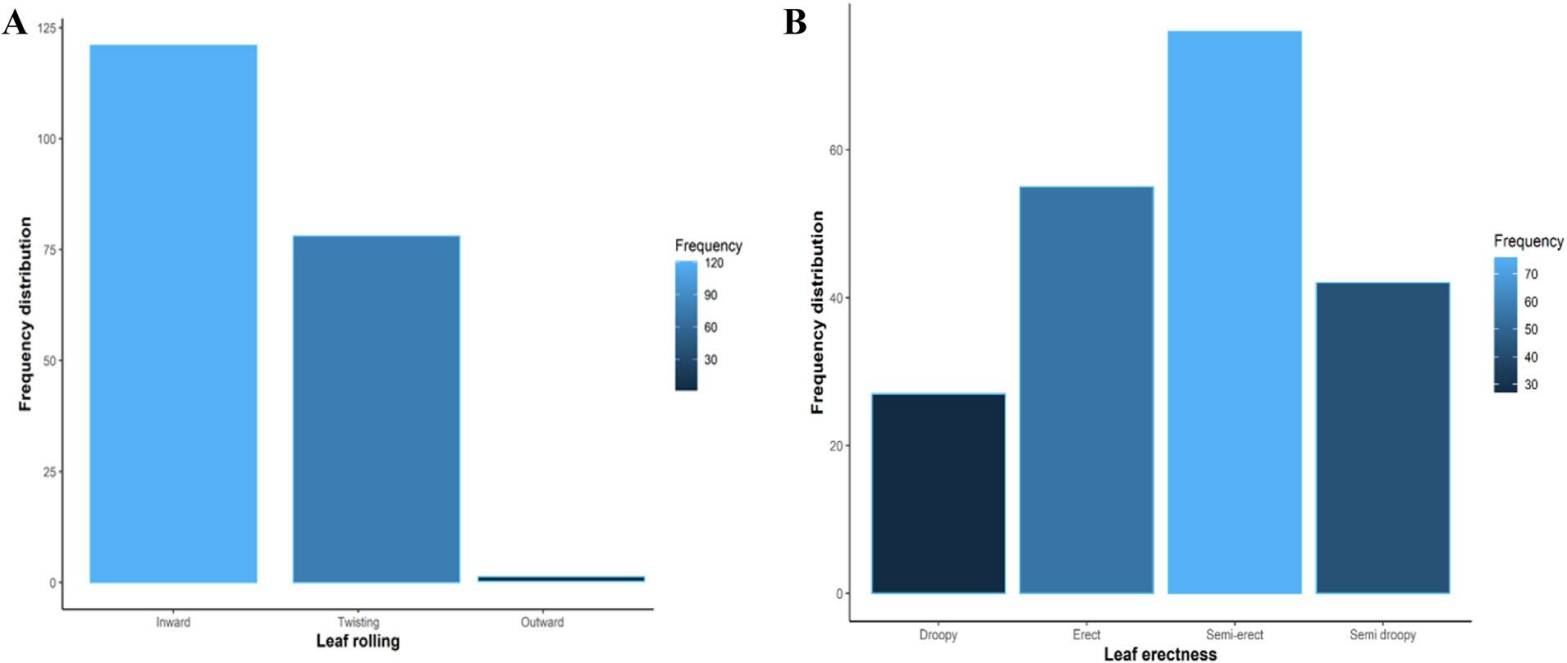
Genotypic frequency distribution of 200 wheat genotypes for leaf surface traits i.e. leaf rolling (**A**) and leaf erectness (**B**)

The phenotypic data analysis of variance showed significant (P < 0.001) differences for leaf rolling and leaf angle among the 200 wheat genotypes under normal growing conditions. Magnitude of genotypic coefficient of variation was lower than phenotypic and indicated that variability also influenced by environmental factors. Broad sense heritability was 97% for leaf rolling and 96% for leaf angle (Table 1). The high broad-sense heritability indicated that genetic factors influenced leaf rolling and leaf angle variation in the genotypes. These results confirms the existence of variability in the genotypes for leaf traits and also offers the chances of selection and improvement. The frequency distribution of leaf rolling and leaf angle are presented in Fig. 2. The maximum number of genotypes had inward leaf rolling and semi-erect type leaf angle. Phenotypic combinations of leaf rolling (inward and twisting type) with leaf angle were observed and divided into seven different combinations. Seventy-two, genotypes were selected from each combination for the evaluation of fog capturing (Additional file 1: Table S2).

**Table 1:** Analysis of variance (ANOVA) and variability parameters of the leaf traits rolling and leaf angle

Traits	Mean sum of square			Coefficient of variation		Broad sense heritability
	Replication (df = 1)	Genotypes (df = 199)	Error (df = 199)	PCV (%)	GCV (%)	h^2^ (%)
Leaf rolling	0.022	0.497 ***	0.007	19.39	19.100	97
Leaf erectness	0.01	1.916 ***	0.035	35.09	34.45	96

*PCV *phenotypic coefficient of variation, *GCV* genotypic coefficient of variation

*** indicates significant difference at P < 0.001

**Fig. 3:**
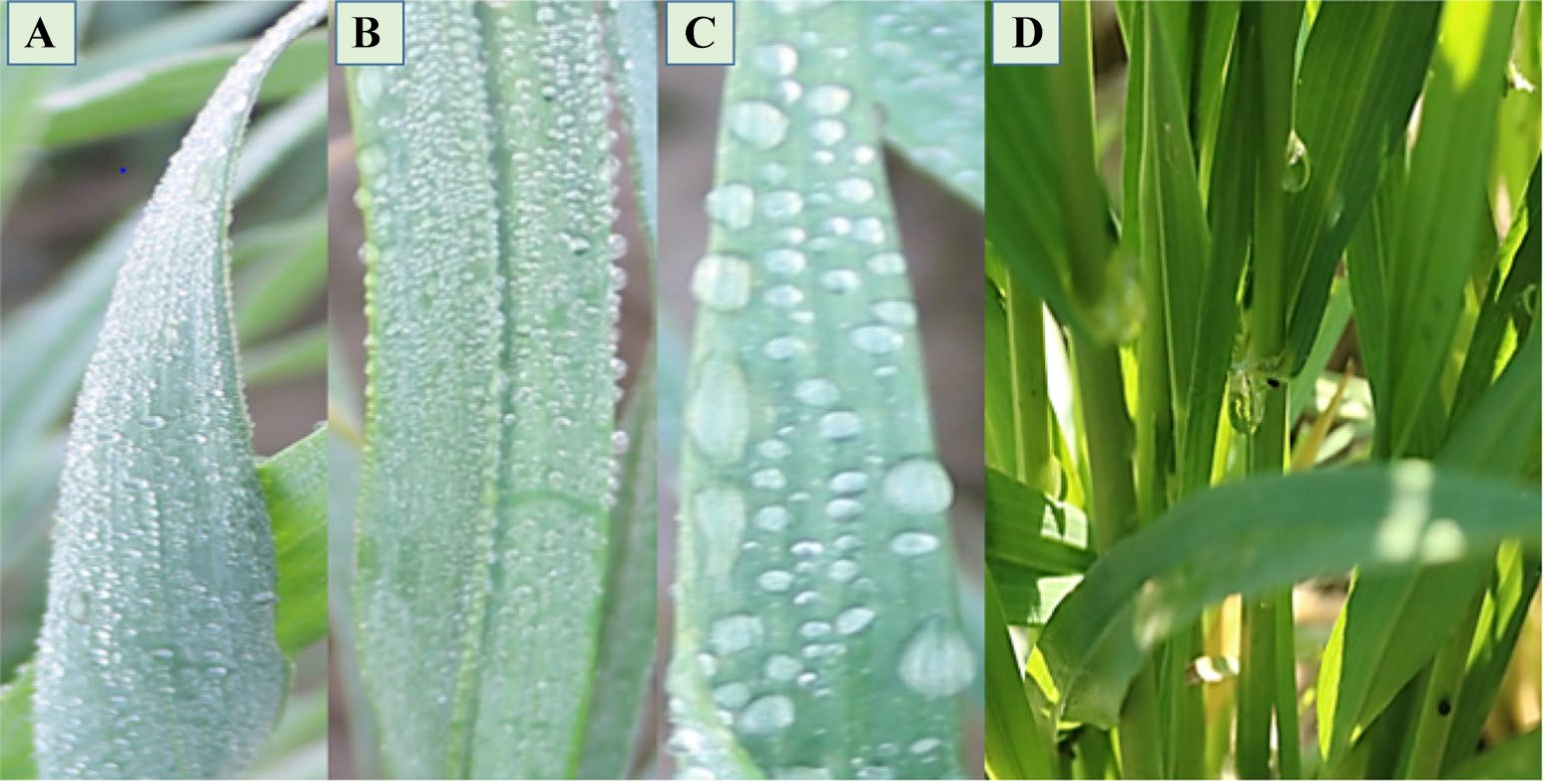
Fog capturing and transport of droplets, fine droplets on **A** abaxial and **B** adaxial leaf surfaces, **C** progressive droplet growth and coalesce with each other, and **D** moving toward the base by stem flow

### Fog capturing and phenotypic combinations

**Fig. 4:**
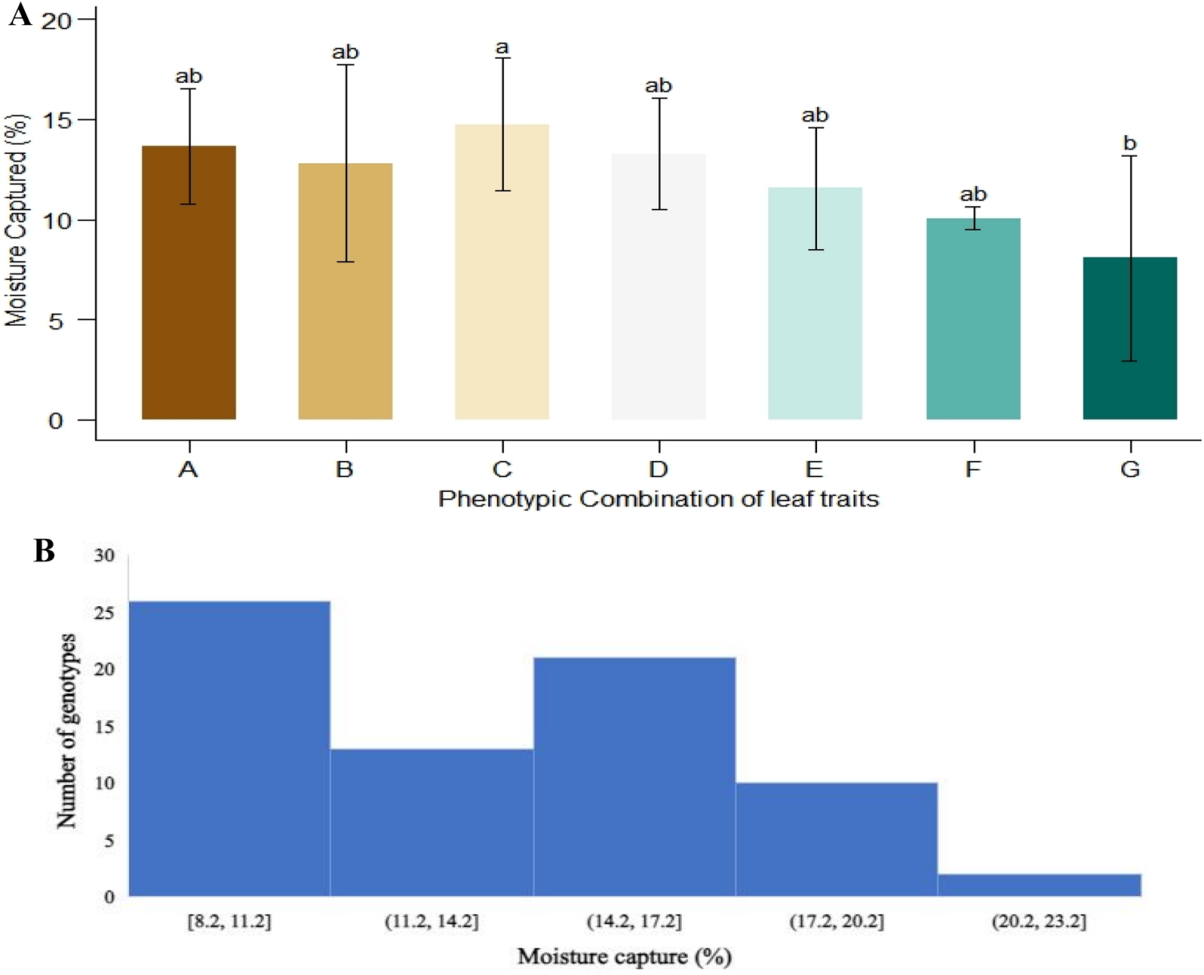
The moisture capture (%) by phenotypic combinations i.e. **A** Inward and erect, **B** Inward, outward and erect, **C** Inward and semi-erect, **D** Inward, outward and semi-erect, **E** Inward and semi-droopy, **F** Inward, outward and semi-droopy and **G** Inward and droopy). Results are presented as mean and bar with the different letters are significantly different according to Tukey's HSD of leaf traits (A). Histogram (B) shows the frequency of no. of genotypes into the each interval of moisture captured

During fog events, it was observed that the leaves of the wheat plants were covered with fog water droplets. Droplets growth was observed at the leaf surface whereby, fine droplets nucleate at the leaf surface, edges and tips. Then droplets coalesce to form bigger droplets and moves through longitudinal grooves towards the base of the leaf. Inward and twisting type rolling and erect angle of leaf base with stem helps in the retention of droplets within the root-zone of the plant (Fig. 3). It was observed that the upper soil of the plant was wet due to fog water inputs (Figs. 1 and 4). For the measurement of fog capturing, 72 wheat genotypes were evaluated for moisture contents within the root zone of the plant and vicinity. Means of the moisture percentage to screen out the superior combinations of phenotypic traits were presented in Fig. 4 and S3. It was revealed that genotypes had inward or twisting type rolling with erect and semi-erect angle, capture more than 12% water within the root-zone than vicinity. Genotypes were divided into three groups based on moisture captured percentage i.e. > 15%, > 10%, and < 10%, and 44 genotypes were selected for further evaluation presented in Additional file 1: Table S4.

### Performance of wheat genotypes under drought stress

**Fig. 5:**
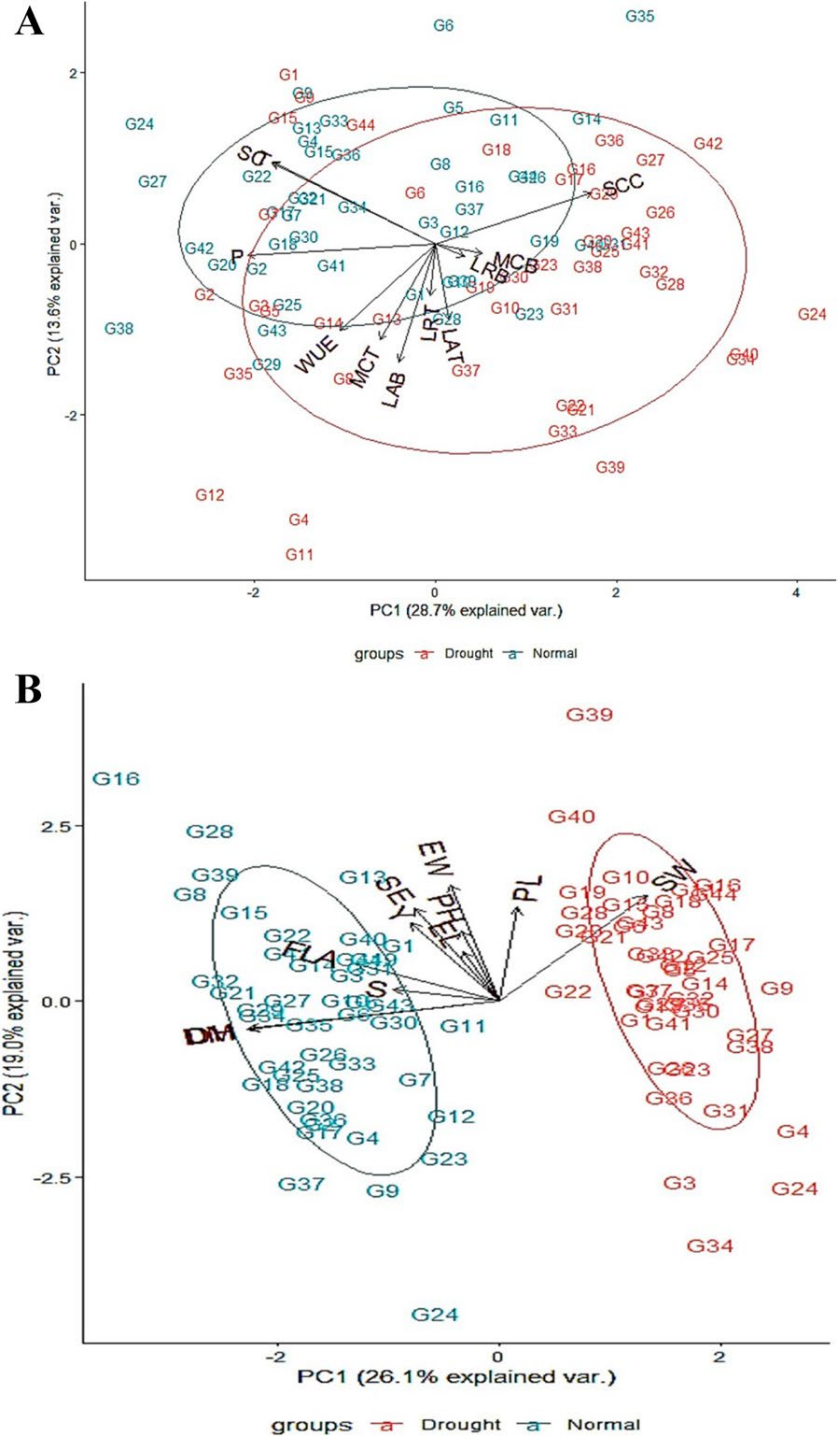
Biplot analysis of **A** leaf traits i.e. leaf rolling (LRT and LRB), leaf angle (LAT and LAB) at tillering (T) and booting (B), moisture capturing (MC), physiological parameters i.e. stomatal conductance (SC), sub stomatal conductance (SCC), photosynthesis (P), transpiration (T) and water use efficiency (WUE), and **B** yield parameters i.e. flag area (FLA), peduncle length (PL), ear length (EL), plant height (PH), days to heading (DH), days to maturity (DM), ear weight/spike (EW), seed weight/spike (SW), spikelets/spike (SS), no. of seed/spike (SE) and yield/plot (Y) of 44 wheat genotypes. Arrows show the magnitude of the traits for their respective environment. The ellipse shows the groups of normal and drought added by the default confidence interval of 68%

Selected wheat genotypes were further evaluated under drought stress conditions for leaf traits, fog capturing, and physiological parameters. Biplots were constructed for the assessment of genotypes in normal and drought fields and variation indicated by PC1 (28.7%) and PC2 (13.6%) for leaf traits, moisture captured, and physiological parameters (Fig. 5). The trait biplot showed a strong correlation between LR and MC at both growth stages as there was a very low angle between their corresponding lines. Although a lower angle was also found between LA and WUE, the variations were lower for LR due to smaller corresponding lines. Stomatal conductance and transpiration, on the other hand, had a strong negative association with LR and MC at the booting stage as the angle between their corresponding lines was greater. Under normal conditions, biplot analysis showed that G20 (14.28%), G29 (7.14%), G42 (14.28%), and G38 (11.42%) were the most moisture captured genotypes at the tillering stage while G6 (12.85%) at the booting stage. However, under drought, G2 (14.28%), G4 (21.42%), G11 (14.28%), G12 (20%), and G35 (7.14%) captured more moisture than others at tillering stage while G24 (14.28%) at the booting stage. The biplot of yield contributing traits explained variation 26.1% and 19% by PC1 and PC2 respectively. The trait biplot showed a strong correlation between GY and other studied traits except for SW and DM. Genotype G8, G16, G28 and G39 under normal conditions while G39 under drought, were more yielded genotypes than others.

**Fig. 6:**
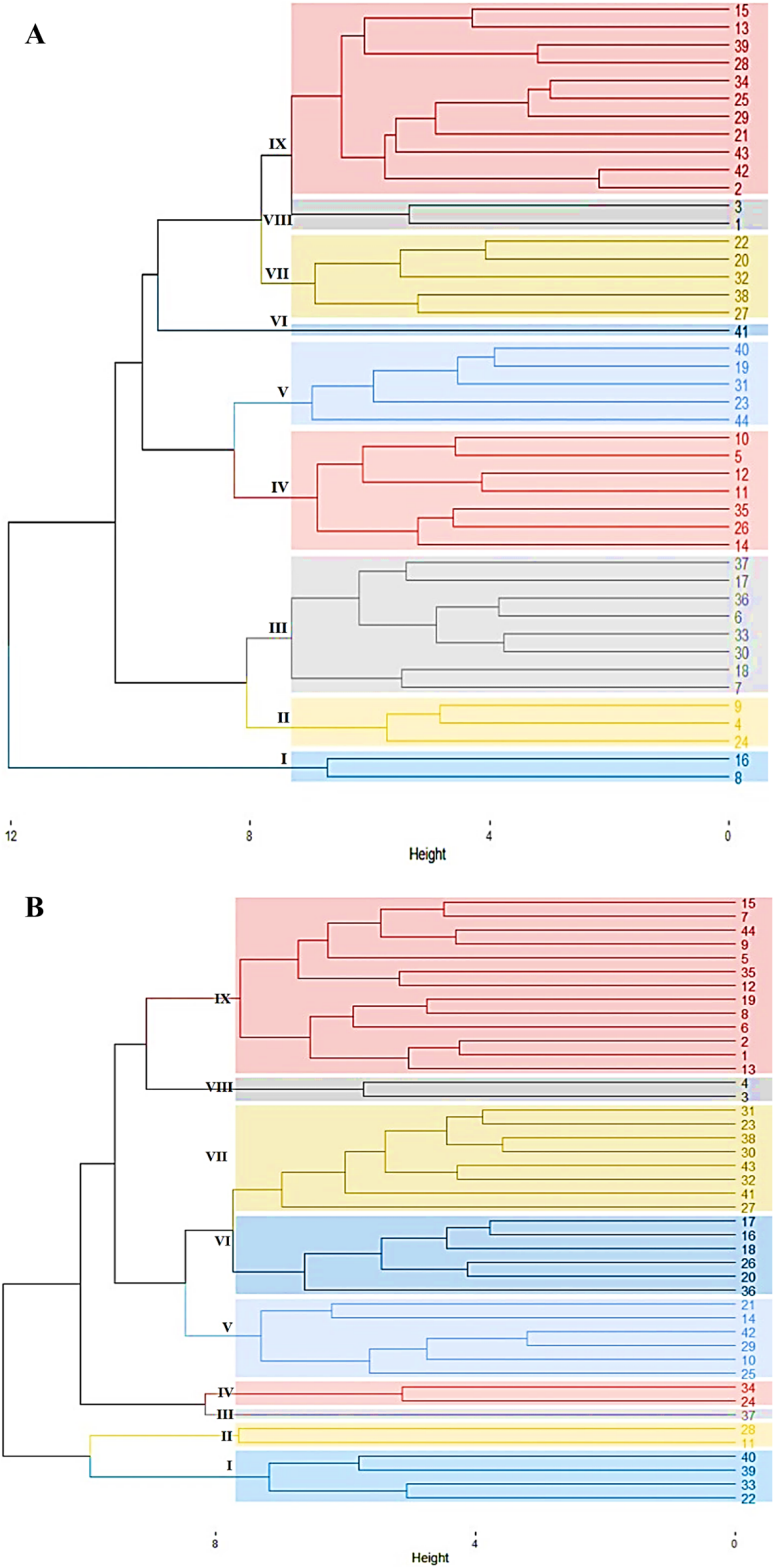
Cluster analysis of 44 wheat genotypes under normal (**A**) and drought (**B**) field conditions

Cluster analysis was used to classify genotypes based on their fog capturing performance under normal and drought stress conditions. Cluster analysis categorized genotypes into nine classes under both conditions (Additional file 1: Table S5 and Fig. 6). In normal conditions, class 8 consisted of two genotypes that performed best for fog capturing (average 14.29%) at tillering stage while at booting class 1 was the more fog captured (average 3.57%). Genotypes in class 6 also performed superior for water use efficiency and yielding parameters. Similarly, under drought stress conditions, class 8 also has the highest percentage of fog capturing (average 17.86) at tillering stage while class 2 performed superior for water use efficiency and fog capturing with an average value 14.29% at the booting stage. Similarly, class 3 has the highest mean values for most of the yielding traits.

### Association of leaf traits, moisture capturing, physiological parameters, and yield contributing traits

**Fig. 7:**
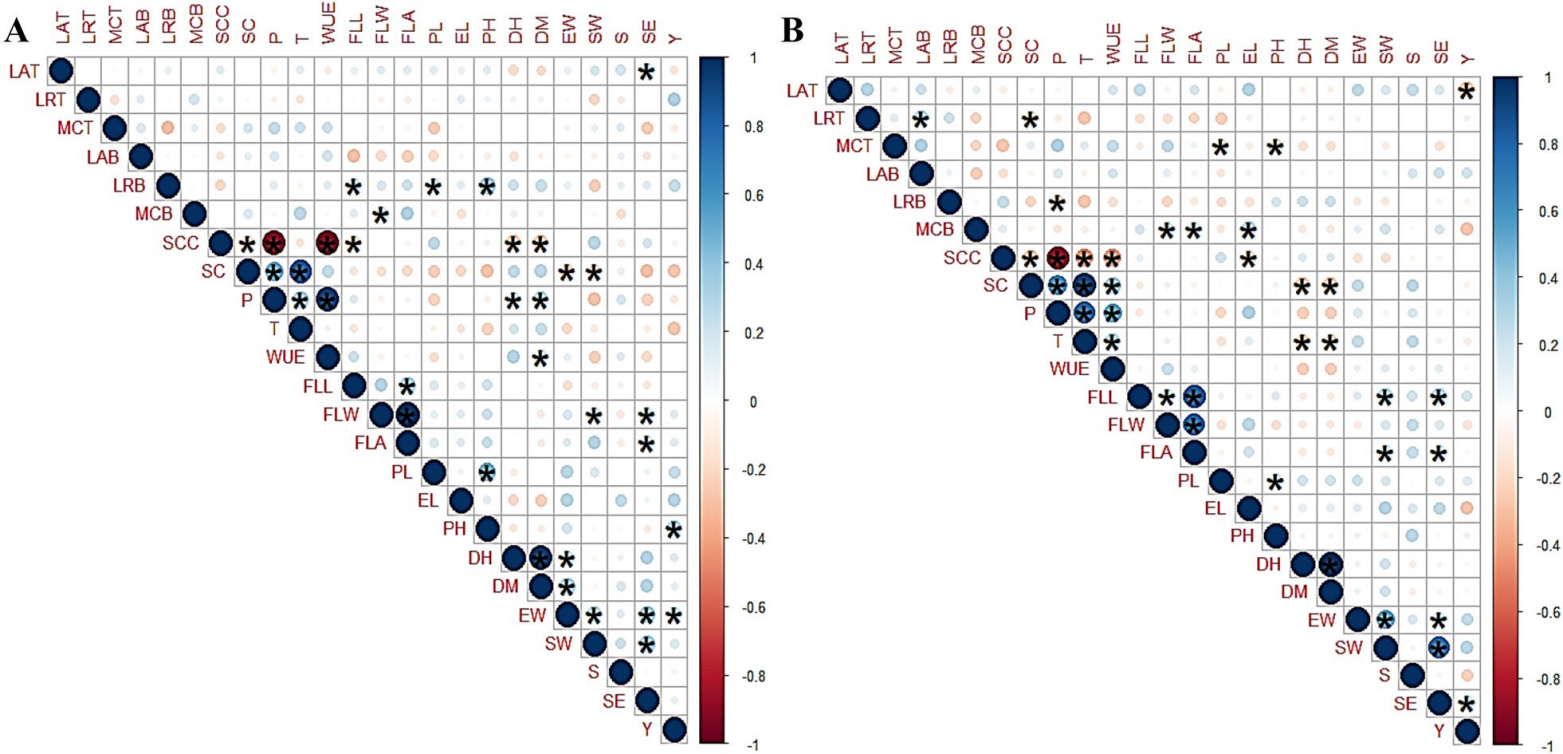
Corplot of phenotypic correlation among leaf traits viz leaf rolling (LRT and LRB), leaf angle (LAT and LAB) at tillering (T) and booting (B), moisture capturing (MC), physiological parameters viz Stomatal conductance (SC), sub stomatal conductance (SCC), photosynthesis (P), transpiration (T) and water use efficiency (WUE), and yield parameters viz flag leaf length (FLL), width (FLW) and area (FLA), peduncle length (PL), ear length (EL), plant height (PH), days to heading (DH), days to maturity (DM), ear weight/spike (EW), seed weight/spike (SW), spikelets/spike (SS), no. of seed/spike (SE) and yield/plot (Y) of 44 wheat genotypes under normal (**A**) and drought (**B**) field conditions. The blue shade shows a positive correlation and the pink shade shows a negative correlation. The size of the circle shows how traits are associated with each other. More size means strong association. * indicates significant (P < 0.05) and without sign (*) indicates non-significant (P ≥ 0.05)

Information on associations between leaf traits, moisture capturing, physiological parameters, and yield contributing under normal and drought conditions will help in the identification of desirable secondary traits to improve grain yield under both conditions. Corplot of phenotypic correlations between studied traits is presented in Fig. 7. Under normal conditions, moisture captured at the tillering stage was found a positive correlation with moisture captured at booing, physiological parameters (SC, P, T and WUE), and yielding traits (EW, SW, and SS). While moisture captured at booting was also found a positive correlation with physiology parameters (SC, P, T, and WUE) and yield traits (FLL, FLW, FLA, DH and DM). Among leaf traits, leaf rolling at tillering was found to have a significant positive correlation with grain yield/plot and moisture capture at the booting stage. While leaf rolling at the booting stage is positively associated with yielding traits (FLL, PL, and PH). Similarly, leaf angle at the tillering stage was found a positive correlation with LRB, SSC, T, FLA, PL, EL, PH, EW, SW, SS, and SE. While, at booting, a positive correlation was found with LRB, P, WUE, FLW, EL, SW, SS, SE, and GY. Grain yield was found to have a significant positive correlation with PH and EW.

Under drought stress conditions, moisture captured was found a positive correlation with WUE, LRT, and LAT but negatively correlated with yielding parameters at tillering stage, while at booting positively correlated with FLW, FLA, EL, EW, SW, and SE. Among leaf traits, leaf rolling at tillering was found to have a significant positive correlation with LAB while a significant negative with P at booting. Similarly, leaf angle at tillering was found positively correlated with LRT, MCT, MCB, LAB, WUE, FLL, FLA, EL, EW, SW, SS, and SE while at booting positive correlation was found with LRB, P, WUE, FLW, SS, SE, and GY. A significant negative correlation was also found between GY and LAT. Among yielding traits, only SE was found to have a significant positive correlation with GY.

### Leaf surface wettability and its association with leaf traits and physiological parameters

**Fig. 8:**
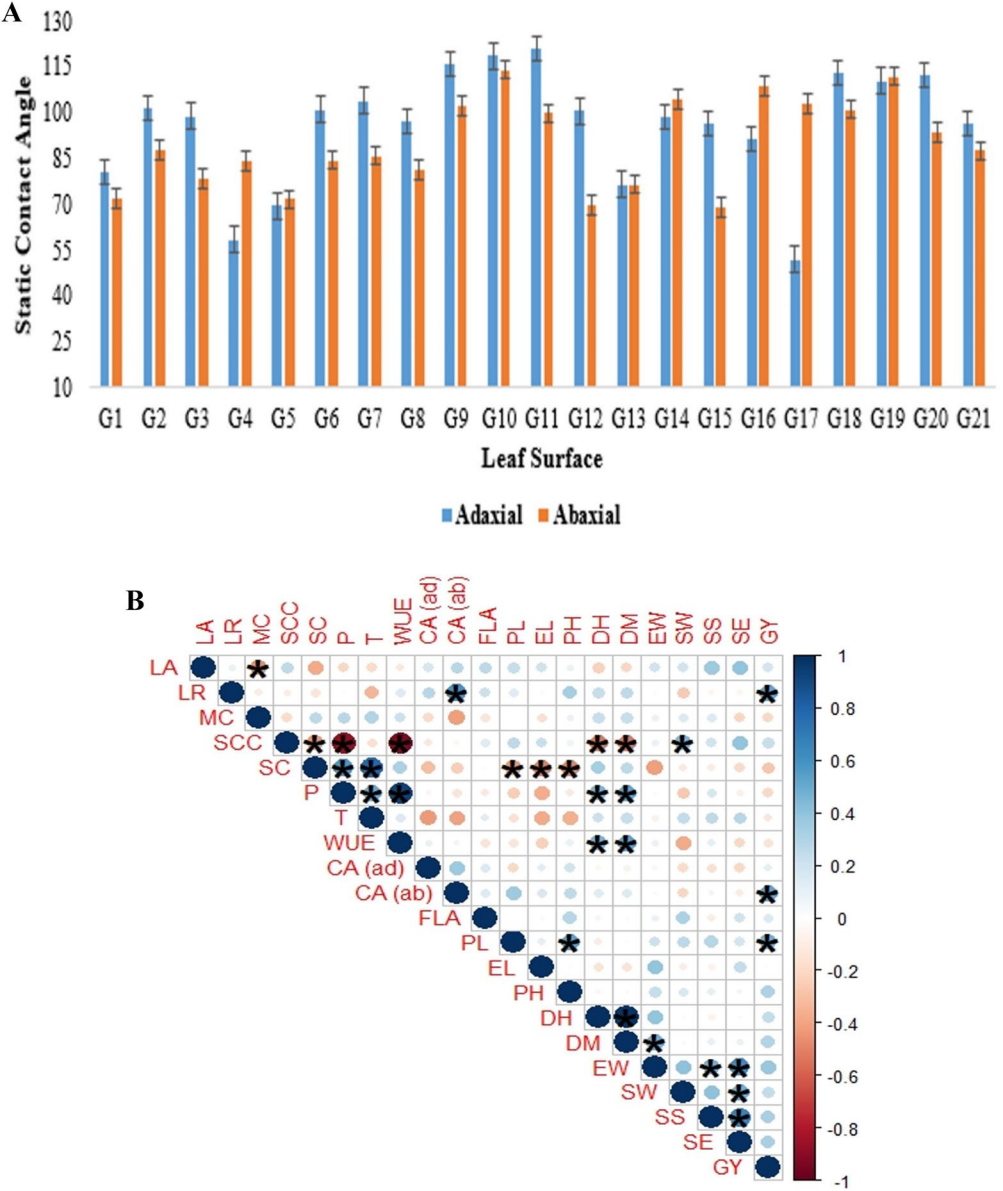
**A** The static contact angle of the abaxial and adaxial leaf surface of 21 genotypes. Results are presented as mean ± SEM (standard error of the mean). **B** Corrplot of leaf traits i.e. leaf rolling (LR) and leaf angle (LA), moisture capturing (MC), physiological parameters i.e. substomatal conductance (SCC), stomatal conductance (SC), photosynthesis (P), transpiration (T), and water use efficiency (WUE), and contact angle (CA) i.e. adaxial (ad) and abaxial (ab). The blue shade shows a positive correlation and the pink shade shows a negative correlation. The size of the circle shows strength of association with each other. * indicates significant (P < 0.05) and without sign (*) indicates non-significant (P ≥ 0.05)

The static contact angle of the abaxial and adaxial leaf surface of 21 selected genotypes is presented in Fig. 8. The abaxial and adaxial surfaces of the leaf showed different wettable properties. The hydrophobic surfaces (> 90° CA) formed spherical drops while hydrophilic surfaces (< 90° CA) spread the droplets. The leaf surface of G9, G10, G11, G18, G19, and G20 were hydrophilic for both abaxial and adaxial surfaces. Some genotypes show the dual wettable properties presented in Fig. 8A.

The corrplot of leaf traits, moisture capturing, physiological parameters, and contact angle are presented in Fig. 8B. Leaf rolling and leaf angle had a positive association with the contact angle of both surfaces (adaxial and abaxial). Moisture capturing had a negative association with contact angle. Physiological parameters also show the same trend of association except for WUE. Leaf surface wettability, moisture capturing and leaf rolling were positively related to WUE. Yielding traits show positive correlation with LA except DH and DM. Similarly, LR also show positive association except SW, SS and SE. Among physiological parameters, WUE show positive association with PH, DH, DM, EW and SS. Moisture content also show positive association with most of yielding traits except FLA, EL, SE and GY. Contact angle of adaxial leaf surface show the positive association with most of the yielding traits except SW, EW, SS and SE.

## Discussion

Fog plays a relevant ecological role as an alternative water source for plants (Burgess and Dawson 2004; Goldsmith et al. 2013; Malik et al. 2014). Wheat plants possess the leaf morphology of efficient fog capturing analogues to other plants as presented by (Azad et al. 2015; Ju et al. 2012; Roth-Nebelsick et al. 2012; Sharma et al. 2018, 2016). Twisting type leaf rolling i.e. cone-shaped leaf helps the plant in the leaf water movement as similar in *Stipagrostis sabulicola* (Roth-Nebelsick et al. 2012), movement of the water droplets to the root-zone further enhanced by the leaf angle. These wheat leaf traits also have an important role in drought tolerance. Leaves are the main site of photosynthesis, and their three-dimensional architecture depends on water availability (Barnabás et al. 2008; Burkhardt et al. 2012; Rebetzke et al. 2001). The exploitation of the wetting mechanism in wheat can increase the water status of plants by morphological features such as leaf rolling and leaf erectness. The present study focused on the phenotyping of wheat genotypes for leaf traits, morpho-physiological, and yield contributing traits to assist the breeding of climate-smart wheat.

Climatic data showed that Multan regions had fog events when the wheat crop is at the vegetative growing stage (Additional file 2: Figs. S1 and S2). Real-time data of climatic parameters recorded by the weather station revealed that December, January, and February were the months when atmospheric air becomes saturated with water vapors and moisture. Relative humidity and leaf wetness were also maximum at that time.

In this study, genotypes were characterized for leaf traits i.e. leaf rolling and leaf angle. Results of variability show that leaf rolling is an environmentally controlled trait while leaf angle is under genetic control. The significant variation among genotypes for leaf traits is indicated by analysis of variance (Table 1). Different phenotypic combinations show that genetic diversity of leaf traits were exist for fog capturing in wheat. Inward and twisting type leaf rolling with erect and semi-erect leaf angle capture more water (< 12%) within the root zone (Fig. 4). Wheat plants can change their phenotype in response to drought stress similar findings were also found in our results (Richards et al. 2010). Genotypes show variation for all the studied traits under both conditions (Fig. 5). It reveals that leaf traits changings with the change in environmental conditions as well as growth stages (Rebetzke et al. 2001; Rosado and Holder 2013).

Under drought stress, the biplot showed a strong correlation between LR and MC at both growth stages. Genotypes (i.e. G2 and G11 at tillering and G24 at booting) having phenotypic combination inward and twisting type rolling with semi-erect angle captured more water within the root-zone. These genotypes are also called fog capturing genotypes (Figs. 5 and 7). In the previous study, it was found that leaf rolling expression depends on the evaporative demand and heterogeneity of soil water content (Rebetzke et al. 2001).

Physiological parameters, development of leaf traits, and soil moisture variation ultimately affect the growth and yield under drought stress. But many times their reliability for effective phenotyping remains doubtful (Eller et al. 2013; Liang et al. 2002; Sarto et al. 2017; Werker 2000). In the present study, the association of leaf traits LR and LA was observed to have a positive correlation with the fog capturing at tillering stage. Thus, inward and erect type canopy of wheat genotypes (G11 and G24) at tillering stage captured fog water. Similarly, LR and LA at booting were observed to have positive and negative correlations respectively and show that inward and semi droopy type canopy (G11 and G24) were linked to fog capturing (Fig. 7). LR was positively correlated with grain yield/plot at tillering stage, revealed that inward rolling contributes more while twisting type rolling correlated at booting stage under stress conditions. A similar trend of LA was observed indicating that droopy leaf angle at tillering and erect type angle at booting can increase grain yield under stress conditions. Among physiological parameters, especially water use efficiency was positively correlated with yield contributing traits. The physiological parameters were negatively correlated with leaf rolling. It means that inward rolling causes the reduction of photosynthesis, transpiration, and water use efficiency. However, physiological parameters were positively associated with moisture capturing at both stages indicating that fog capturing genotypes also had higher photosynthesis and water use efficiency. Genotypes with higher water use efficiency (Fischer et al. 1998; Sarto et al. 2017) are higher-yielding under water stress conditions.

Positive association of leaf traits with contact angle indicated that inward and erect type canopy is a morphology often associated with the hydrophobic property (Fig. 8). It shows that the wetting mechanism in wheat takes place through leaf water repellency and stem flows that eventually increased the soil moisture within the root zone (Rosado and Holder 2013). Leaf wetting has a positive effect on plants as it prevents water losses through transpiration and improves water status, growth, and survival (Berry et al. 2019; Eller et al. 2013; Goldsmith et al. 2013; Gürsoy et al. 2017). Leaf water repellency, the interaction of a water droplet with the leaf surface, is a function of leaf composition, and structure (Neinhuis et al. 2001). The negative association of leaf wettability, leaf trait, and transpiration indicates that the leaf hydrophilicity in wheat is associated with high moisture capturing efficiency provided that the plant exhibits inward type leaf rolling and erect stature. Wheat genotypes captured a maximum of 12–20% water which is enough to fulfill the water requirements (Fig. 4). Moreover, leaf surface wettability (hydrophilic property/repellency) further enhances fog capturing mechanism in wheat. Positive association of leaf surface with yielding parameters also revealed that fog capturing in wheat may enhance the yield in wheat. In short, these findings supported that self-irrigated wheat can produce good yield in semi-arid regions.

## Conclusion

In the arid and semi-arid region, fog harvesting/capturing is an alternative source of water for plants. This study reveals that increased wettability (hydrophilicity) combined with leaf traits (inward to twisted and erect to semi-erect angle) leads to enhanced fog capturing in wheat. Diversity of leaf traits i.e. leaf rolling and leaf angle also existed in wheat that would be utilized for the development of self-irrigated wheat. Genotypes having these leaf features would be better adapted under water stress conditions and produce good yield However, the regulatory mechanism of leaf rolling and leaf angle may also need to be studied further in contrasting environments.

## Supplementary Information

**Additional file 1. Table S1.** Plant material consisted of 200 genotypes in this study. **Table S2.** Phenotypic combinations and number of selected genotypes in each combination. **Table S3.** Means values of moisture captured by selected genotypes. **Table S4.** Grouping of the genotypes on the basis of moisture captured percentage. **Table S5.** Mean values of classes of wheat genotypes under normal and drought conditions. **Table S6.** Mean data of genotypes used for statistical analysis.

**Additional file 2: Fig. S1**. Historic climatic pattern of Multan region (2008 to 2017). **Fig. S2**. Daily average climatic parameters data during wheat-growing season (2018-19).

## Data Availability

The datasets generated during and/or analyzed during the current study are available in supplementary table file.

## Abbreviations

DAS: Days after sowing
AWS: Automatic weather station
MC: Moisture captured
RZ: Root-zone
V: Vicinity
MSg: Mean square of genotype
MSe: Mean square of random error
δ^2^g: Estimated genetic variance
δ^2^e: Estimated error variance
δ^2^p: Estimated phenotypic variance
H^2^: Broad sense heritability
LR: Leaf rolling
LA: Leaf angle
T: Tillering
B: Booting
SC: Stomatal conductance
SCC: Sub stomatal conductance
P: Photosynthesis
T: Transpiration
WUE: Water use efficiency
FLL: Flag leaf length
FLW: Flag lead width
FLA: Flag leaf area
PL: Peduncle length
EL: Ear length
PH: Plant height
DH: Days to heading
DM: Days to maturity
EW: Ear weight/spike
SW: Seed weight/spike
SS: Spikelets/spike
SE: No. of seed/spike
Y: Yield/plot
Ad: Adaxial
Ab: Abaxial
CA: Contact angle
