# Supplementary material for: Leaf rolling and leaf angle improve fog capturing and transport in wheat; adaptation for drought stress in an arid climate
Source: Bot Stud. 2022 May 16;63:13. doi: 10.1186/s40529-022-00343-y (PMC9110575; doi:10.1186/s40529-022-00343-y)
Supplement: Supplementary file 2 — Additional file 2: Fig. S1. Historic climatic pattern of Multan region (2008 to 2017). Fig. S2. Daily average climatic parameters data during wheat-growing season (2018-19). [file 40529_2022_343_MOESM2_ESM.docx]

**Leaf rolling and leaf angle improve fog capturing and transport in wheat; adaptation for drought stress in an arid climate**

Sabah Merrium^1^, Zulfiqar Ali^1^,*, M. Habib-ur-Rahman^2,3^,*, Sadia Hakeem^1^ and Muhammad Arslan Khalid^1^


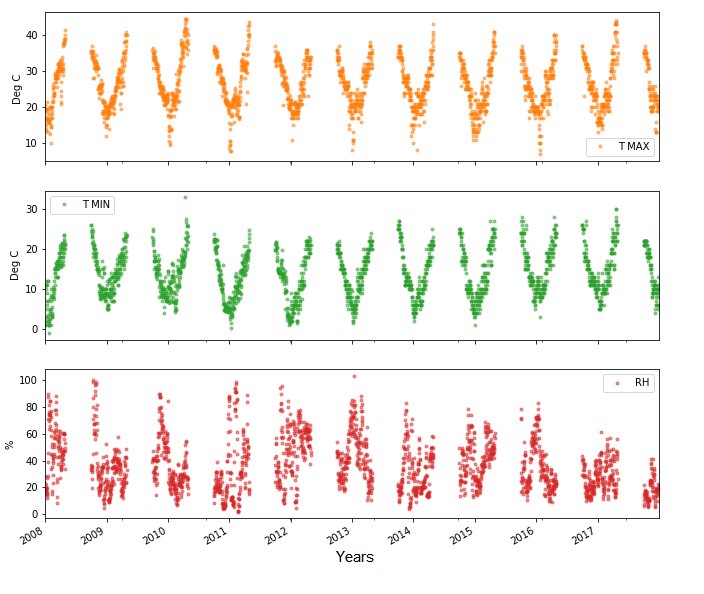


**Fig S1.** Historic climatic pattern of Multan region (2008 to 2017).


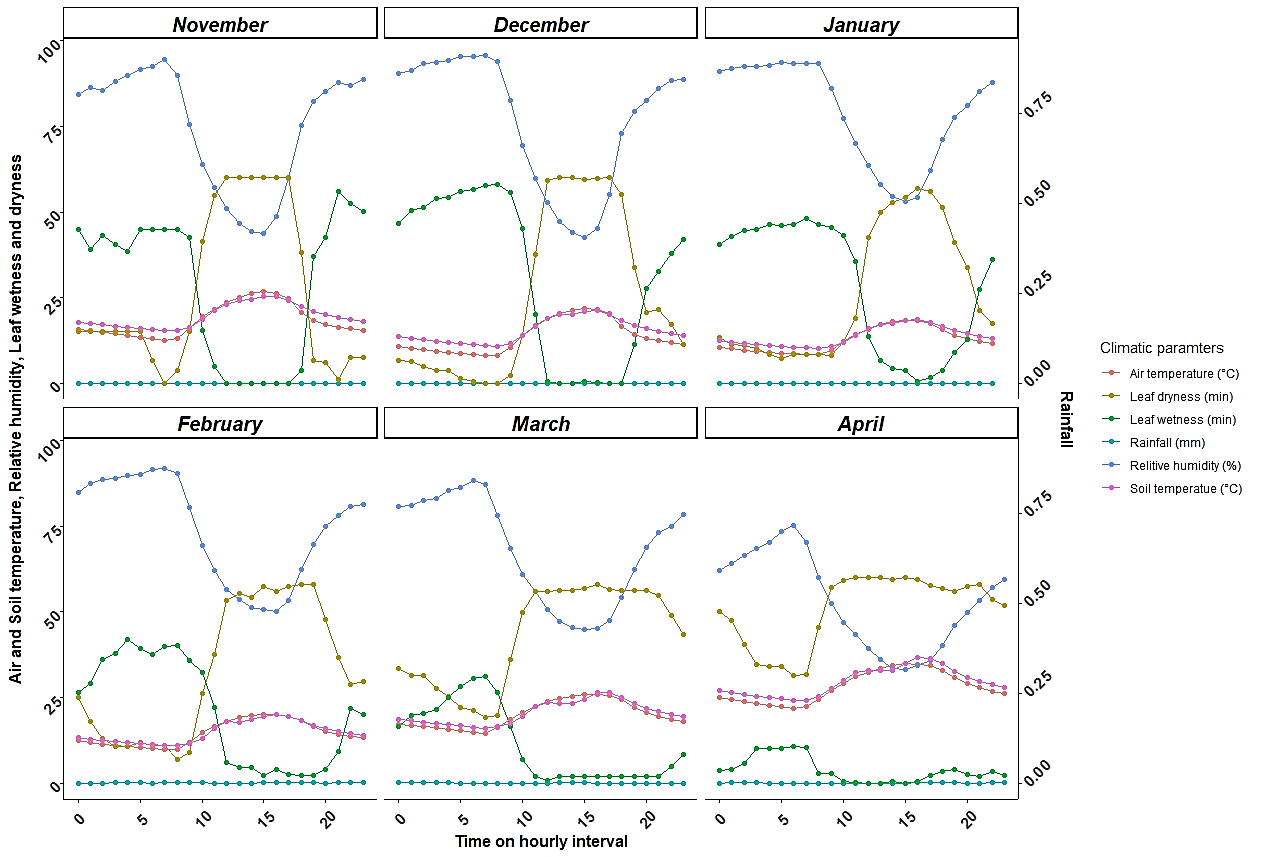


**Fig S2.** Daily average climatic parameters data during wheat-growing season (2018-19).
